# Book Notices

**Published:** 1880-08-20

**Authors:** 


### *BOOK NOTICES.*

THE SURGERY, Surgical PATHOLOGY and Surgical ANATOMY of the FEMALE PELVIC ORGANS, in a series of plates taken from nature, with commentaries, notes and cases, by Henry Savage, M.D., Lond., Fellow of the Royal College of Surgeons of England, etc. Third edition, revised and greatly extended ; 32 plates and 22 wood engravings, with special illustrations of the operations of vesico-vaginal fistula, ovariotomy and perineal operations.—New York, Wm. Wood & Co., 27, Great Jones Street, 1880.

This is one of the most instructive and useful works of the series of standard authors being issued by Wm. Wood & Co. The illustrations are superior to anything we have seen in gynecological works heretofore published.

NASO-PHARYNGEAL CATARRH, by Martin Coomes, M.D., Professor of Physiology, Ophthalmology and Otology in the Kentucky School of Medicine; Member of the American Medical Association; of the Kentucky State Medical Society, etc., etc.—Louisville, Kentucky, Bradley & Gilbert, Publishers, 1880.

This is an illustrated work of 165 octavo pages, in plain type, neatly printed. It is practical and must prove exceedingly useful, containing much valuable information for the practitioner.

TREATISE ON THERAPEUTICS: Translated by D. F. Lincoln, M. D., from the French. A. Trosseau, Prof. of Therapeutics in the Faculty of Medicine of Paris, Physician to Hotel Dieu, etc., etc., and H. Pidoux, Member of the Academy of Modern Honorary Physicians to Hospitals. Ninth Edition. Revised and enlarged with the assistance of Constantine Paul, Prof. of Agrege in the Faculty of Medicine, Paris, etc. Vol I. New York, William Wood & Co., 27 Great Jones St., 1880.

A work of 302 octavo pages, plain, practical and easy to be understood. Useful to the practitioner, and especially so to the student of medicine.

THE STUDENT'S DOSE-BOOK AND ANATOMIST COMBINED. By C. HENRI LEONARD, A.M., M.D., Professor of Medical and Surgical Diseases of Women and Clinical Gynæcology in Michigan Medical College, etc.

Part I. The Multum in Parvo Reference and Dose-Book, third revised edition.
Part II. The second revised edition. Price $1.00. Detroit: 1880.

